# Occupational complexity of paid work and housework, and its impact on the cognitive performance in community dwelling older adults, preliminary results

**DOI:** 10.1590/1980-5764-DN-2023-0038

**Published:** 2024-03-11

**Authors:** Carolina Feldberg, Juan Pablo Barreyro, Maria del Rosario Quián, Paula Daniela Hermida, Silvia Deborah Ofman, Natalia Carolina Irrazabal, María Florencia Tartaglini, Cecilia Serrano

**Affiliations:** 1 Instituto de Neurociencias Buenos Aires, Consejo Nacional de Investigaciones Científicas y Técnicas, Capital Federal, Buenos Aires, Argentina.; 2 Universidad de Buenos Aires, Facultad de Psicología, Consejo Nacional de Investigaciones Científicas y Técnicas, Capital Federal, Buenos Aires, Argentina.; 3 Instituto de Neurociencias Buenos Aires, Capital Federal, Buenos Aires, Argentina.; 4 Universidad de Palermo, Facultad de Ciencias Sociales, Consejo Nacional de Investigaciones Científicas y Técnicas, Capital Federal, Buenos Aires, Argentina.; 5 Hospital César Milstein, Servicio de Neurología Cognitiva, Capital Federal, Buenos Aires, Argentina.

**Keywords:** Aging, Activities of Daily Living, Cognition, Occupations, Envelhecimento, Atividades Cotidianas, Cognição, Ocupações

## Abstract

Work and activity could be an important source of cognitive enrichment. Activities that are more challenging concerning the cognitive functions that are put into practice are associated with lower risk of cognitive decline in old age.

**Objective:**

The present study aimed to assess the impact of occupational complexity and household tasks in three cognitive domains (verbal episodic memory, language, and executive functions) in older adults residing within the community.

**Methods:**

A trail analysis was executed, using the structural equations procedure in 120 participants assessed with main lifetime occupational activity and household tasks questionnaire, as well as a neuropsychological assessment battery for memory, language, and executive functions.

**Results:**

The regression weights analysis indicated that complexity in household chores showed moderate effects on executive functions (β=0.19; p=0.027) and that occupational complexity of paid work showed effects on memory (β=0.26; p=0.008), language (β=0.38; p<0.001), and executive functions (β=0.55; p<0.001).

**Conclusion:**

Paid work promotes cognitive reserve, contrary to household activities which seem to have a moderate impact on cognition. Differences in activity complexity not only impact people´s economic and social status and possibilities but can also determine different courses of aging and cognitive risk.

## RESUMO

Trabalho e atividade podem ser importantes fontes de enriquecimento cognitivo. Atividades que são mais desafiadoras quanto às funções cognitivas postas em prática se associam a menor risco de declínio cognitivo em idade avançada.

**Objetivo:**

O objetivo do presente estudo é avaliar o impacto da complexidade ocupacional e das tarefas domésticas em três domínio cognitivos (memória episódica verbal, linguagem e funções executivas) em idosos residentes na comunidade.

**Métodos:**

Foi realizada uma análise de trilha com o uso de procedimento de equações estruturais em 120 participantes, avaliados por meio de: questionário das principais atividades ocupacionais e tarefas domésticas da vida, bem como bateria de avaliação neuropsicológica para memória, linguagem e funções executivas.

**Resultados:**

A análise dos pesos de regressou mostrou que a complexidade nas tarefas domésticas apresenta efeitos moderados nas funções executivas (β=0,19; p=0,027) e que a complexidade ocupacional do trabalho remunerado teve efeitos sobre a memória (β=0,26; p=0,008), linguagem (β=0,38; p<0,001) e funções executivas (β=0,55; p<0,001).

**Conclusão:**

O trabalho remunerado promove a reserva cognitiva, em contraste com as atividades domésticas, que parecem ter impacto moderado na cognição. As diferenças na complexidade das atividades não apenas impactam o status econômico e social e as possibilidades das pessoas, mas também podem determinar diferentes cursos de envelhecimento e risco cognitivo.

## INTRODUCTION

Population aging is a phenomenon that has deepened in recent years generating an increase in the proportion of older adults within the general population. The current challenge for health professionals consists of providing quality to those years that have been won by science in the last decades. One of the problems that most calls for the search for solutions refers to cognitive sphere. Identifying behavioral factors that prevent, reduce, or delay cognitive decline in old age is necessary to develop strategies that improve the quality of life at this stage of the life cycle^1^. There is substantial evidence that suggests that the participation in intellectually challenging activities during middle age is associated with favorable cognitive performance in later life. One of the most important sources of evidence derives from studies conducted on main lifetime occupations and cognitive performance^2^. There is a growing body of research that suggests that more active lifestyles and participation in more complex environments are associated with better cognitive performance in old age^3^ and that the most stimulating environments increase the cognitive reserve of the subject, which later in life could generate a protective effect against the effects of cerebral aging^4^. Therefore, the way we spend our time, whether in work or non-work activities, generates differences between people regarding the possibility of enriching their neural networks, causing disparities to increase in cognitive risk in old age^5^. Leisure activities, including physical, cognitive, and social activities, are major components of modifiable and healthy lifestyles that are beneficial to cognition^6^. Some daily activities appear to provide a form of intellectual stimulation that facilitates the maintenance of normal cognition during the lifespan. Mental exercise provided by frequent engagement in intellectually demanding activities at work may facilitate the maintenance of high levels of neuronal activation which can buffer against neurodegeneration and cognitive impairment in old age^7^. Hakiki et al.^2^ proposed that complex work and leisure environments help to facilitate cognitive performance by motivating people to perform demanding cognitive operations daily.

Previous studies have analyzed the influence that present occupational conditions have on cognitive function. Sörman et al.^8^ observed that regarding executive functions, most enriched and challenging environments demand a more complex type of activity, and produce an increase in cognitive flexibility. The authors also pointed out that simpler work environments decrease these functions^8^. Added to this, Donka & Balogh^9^ found better performance in executive functions in air traffic controllers and professional athletes, respectively, than with subject controls. Therefore, it is worth highlighting that there are several research works that focus on the link between occupational complexity and cognitive functions such as episodic memory, work memory, visual-spatial abilities, and executive functions.

However, less is known about the link that exists between household tasks and cognitive performance. The number of scientific studies that involve subjects who carry out both tasks simultaneously is even smaller. Schooler et al.^10^ were one of the first authors who analyzed housework and its impact on different psychological spheres. They concluded that, in terms of effects, the tasks performed in the field of paid work and household chores would have different psychological consequences^10^. Given the benefits that paid work occupation provides to subjects in relation to cognitive performance and, therefore, the reduction of the risk of developing dementias, it is also significant to analyze the role that the activities performed outside the work environment have on cognitive wellbeing. In a study on leisure time and cognitive performance, Park et al.^11^ noted that housework is one of the main activities performed by Korean older adults. In their results, the authors indicated that there is a negative association between manual domestic chores and cognitive function and a positive association between social activities and voluntary work in low-educated older adults. In a complementary way, Koblinsky et al.^12^ noticed that household physical activities are positively associated with brain volume measurements, specifically gray matter volume but no significant relationships were observed with cognitive measures.

Hence, the objective of this current study was to assess the impact of occupational complexity and household tasks on three cognitive domains (verbal episodic memory, language, and executive functions) in older adults residing within the community.

## METHODS

### Participants

The participants in the study were volunteers over 60 years old who assisted a cognitive neuroscience service at a private institution. Data was obtained within the framework of neuropsychological evaluations of a larger study carried out on patients over 60 years old between August and December of 2021 at a Cognitive Neuroscience Service in Argentina. Starting from an initial database of 634 cases, 120 subjects were selected to participate in the study. Only those who completed all tests, signed the informed consent, and complied with the admission criteria were included in the sample.

The inclusion criteria for participation in this work were minimum age of 60 years and absence of dementia or other neurological illnesses. In addition, all participants should have had paid jobs before retirement and done household tasks at home. The following criteria were used for exclusion of participants: previous diagnosis of depression or other psychiatric disorders meeting the criteria of the Diagnostic and Statistical Manual of Mental Disorders, Fifth Edition (DSM-V) from the American Psychiatric Association^13^, previous diagnosis of probable Alzheimer’s disease or another type of dementia according to the National Institute of Neurological and Communicative Disorders and Stroke and Alzheimer’s Disease and Related Disorders Association (NINCDS-ADRDA) criteria^14^ and DMS-V^13^, cerebrovascular accidents or endocranial neurosurgical interventions, chemotherapy treatment, diabetes, presence of sensory or motor alterations that could interfere with the normal performance of tasks proposals, and the consumption of substances that affect the normal performance of tasks.

This information was corroborated by a neuropsychological evaluation and neurological consultation.

For the selection of the sample, it was considered the scores in the cognitive screening battery, functional level, and cognitive status. The following instruments and criteria were used for the selection of the sample in the present study: the Mini-Mental State Examination (MMSE)^15^ was used for general cognitive status; the study only included subjects with higher values for the cutoff point adjusted by age and education stated by the cited norms; the presence of depression was assessed through the Beck Depression Inventory^16^ and the subject’s score had to be less than 10 points; patient’s functional level was assessed with the Barthel index^17^ and scores were expected to be higher than 60 points in order to include the subject in the sample; instrumental activities of daily life were evaluated through the Inventory of Instrumental Activities of Daily Life (IADL) by Lawton and Brody^18^, where the score should be 0 points, which indicates total functional independence; subjects of the study came from interviews carried out within the framework of cognitive evaluations; and the scales that evaluate the functional level of the patient were answered by their companion, who could only be a family member or acquaintance of the subject being evaluated.

### Ethic statement

The research project was approved by the Teaching and Research Committee of the institution where the project was executed. The study was conducted in accordance with the guidelines established in the International Conference on Harmonization of Technical Requirements for Registration of Pharmaceuticals for Human Use (ICH), the latest revision of the Helsinki Declaration^19^. All participants signed informed consent to take part in the study. All the professionals involved in the study had certification in ethics.

### Materials

It was used an initial screening evaluation which consisted of:

### Sociodemographic data

Information was obtained through a basic data questionnaire, and aspects related to the clinical history and functional level of the patient were investigated. Likewise, a neurological and psychiatric interview was conducted in which the presence of subjective complaints, clinical history, family history of dementia, medication, history of diseases and surgeries, and relevant medical history were explored.

### Neuropsychological evaluation

Episodic memory: Two tests were used:

1. The first was Signoret’s Memory Battery^20^. In this task, the subjects must try to retain and repeat, immediately and in a delayed manner, the greatest amount of data in a story presented verbally. The maximum score is 12 items in total. Two scores are obtained, one corresponding to the immediate recall score from the data recalled by the subjects immediately after the story was read and the other corresponding to what was recalled by the subjects 30 minutes later.
2. The second test was the Verbal Auditory Test Spain-Complutense (TAVEC- *Test de Aprendizaje Verbal España-Complutense*)^21^. It is a verbal learning test that has an initial list (A) of 16 nouns, made up of four different semantic categories. The application consists of five immediate recall trials, followed by an interference list trial (B), which also consists of the same number of words. Subsequently, the subjects are presented with a short-term free recall test from list A and a cued recall test. After an interval of 20 minutes, a delayed free recall test is presented, a recall test with semantic keys and a recognition test, which has 44 nouns. The test allows analyzing, in addition to recall, perseverations and different types of intrusions and false positives.

Language Boston Naming Test^22^: It is a visual confrontation denomination test, made up of 60 drawings of objects to be named after 20 seconds. The subjects are given semantic and later phonological keys. Regarding the score, a summary of spontaneous, latency, and correct responses is obtained from semantic or phonological keys. For the total score, only those produced spontaneously or based on semantic keys are considered correct. The maximum score is 60 points.

Verbal Fluency Test^23^: Letter fluency measures verbal initiation and retrieval skills. Participants spontaneously produced words beginning with given letters across three one-minute trials, and scores are the total number of correct words.

Semantic Fluency Test: This test evaluates the ability to evoke and name words within a certain category in a set time that is usually 60 seconds. The category animals was used for all patients.

Vocabulary Subtest Wechsler Adult Intelligence Scale (WAIS) III^24^: In this test, subjects are asked to define different words that go from frequent to infrequent in their daily use. The total of items presented is 32 and each one can receive a score of 0, 1, or 2 points according to the given answer. The maximum possible score is 66 points. This test reveals the capacity for classification and conceptualization. It begins with item 4 and the administration is interrupted if the subject presents six consecutive responses of 0.

### Executive functions

Trail Making Test (TMT) Part B^25^: TMT-B is a measure of set-shifting and cognitive flexibility. Participants drew lines to connect 25 encircled numbers and letters in alternating order as fast as possible. Scores for the TMT-B were seconds to task completion, with the maximum being 300 seconds.

Digits Backwards^26^: It is a subtest of the Wechsler Memory scale. In this test, the examinees are asked to repeat the series in reverse order, in two trials for each series of digits. The task is interrupted when the subjects obtain a score equal to 0 in both attempts. The maximum possible score in each modality is 16 points.

Analogies Subtest WAIS III^24^: In this task, two elements are presented to subjects orally and they must say how the elements are alike. The total of items presented is 19 and each one can receive a score of 0, 1, or 2 points according to the answer given. The maximum possible score is 33 points. The resolution of this test puts into play the ability to order and classify similar concepts.

Cubes Subtest WAIS III^24^: This is a visual-construction test that also allows for the assessment of execution speed, 26 designs are presented that the patient must reproduce with six-sided cubes that have different patterns. The maximum score is 68 points and extra points are obtained in each item for speed in performance.

Once the instruments yield scores expressed in different scales (normative score, T score, raw score), for comparison and analysis, these are transformed into z-scores. In the neuropsychological field, those scores lower than z=-1.5 are considered altered^27^.

All cognitive test scores herein were transformed into z-scores in order to compare the performance of the subjects in the different tests. The average values obtained are below the cut-off of z=-1.5^27^. All patients included in the study completed all instruments.

Occupational complexity: Questionnaire on Agency of Labor Activity (CAAL — *Cuestionario sobre Agenciamiento de la Actividad Laboral*)^28^. This questionnaire is an adaptation into Spanish of the instrument developed by Kohn and Schooler^29^ and assesses the psychological effects of occupational complexity. It consists of nine open and closed questions with fixed alternatives that assess different aspects related to the main job occupation, including the complexity of the work task. It is evaluated from open questions that are later categorized by the interviewer trained for this purpose, according to the classification grid, where the different levels of occupational complexity are presented for work with different materials (data, people, and things) and the overall complexity of the activity. This classification was elaborated by Kohn and Schooler^29^ on the basis of the Dictionary of Occupational Titles, 3rd edition U.S. Department of Labor^30^. The evaluation of this dimension is carried out through Likert-type scales. The general complexity of the main occupation is assessed on a scale ranging from 1 (task of minimum complexity) to 7 (task of maximum complexity). The time for administering the CAAL is approximately 15 minutes.

Household chores: A translation and adaptation to Spanish of the Household tasks questionnaire developed by Schooler et al.^10^ was used. This instrument is a parallel version of the one developed by Kohn and Schooler^29^ for the workplace, adapted to household tasks. The evaluation of this dimension is done using Likert-type scales. The main general complexity is rated on a scale ranging from 1 (task of minimum complexity) to 7 (task of maximum complexity). The questionnaire inquires about the complexity of working with data, people, and things in the household environment. The questionnaire explores the complexity of household work in each of the dimensions separately: activities that are mainly performed with data, people, and things.

For example, in regard to the complexity of household tasks performed with data, the respondents have to assess if the activities they perform at home are on a scale of complexity that ranges from 1 to 7. The score 1 is for more simple activities and 7 for the more complex ones. The activities with data presented in the questionnaire extend from simple tasks such as reading product labels or writing grocery shopping lists, to more complex ones such as making payments and writing checks, or very complex tasks such as balancing household expenses. Regarding the tasks with people, they are also on a scale from 1 to 7. The respondents are asked about the tasks they do with people in the household. The activities presented also consider simple activities such as serving or helping other family members with everyday tasks or helping others with household chores. Also, other intermediate tasks are asked, such as exchanging information with the objective of accomplishing the work of the household, for example talking with store salesmen, with people who do repairs in the home or domestic help, with the spouse, the children’s teacher. Besides more complex tasks are consulted involving negotiations such as buying a house or borrowing money from the bank for a mortgage. Finally, the questionnaire examines, also on a scale of 1 to 7, the work with things at home from simple manual skills like preparing a sandwich or a cup of tea, to other moderate skills such as using household appliances of different levels of complexity, and even other more complex skills, for example, making gourmet meals or household repairs.

And then an overall score is obtained in which the interviewees are asked to assess their general level of complexity based on the answers given before. The answers are also on a scale of complexity that ranges from 1 to 7. A score of 1 is for more simple activities, and 7 for the more complex ones. This last general score is the one used in the present study.

### Procedures

For the recruitment of subjects, individuals who spontaneously consulted or were referred to the Cognitive Neuroscience Service were invited to participate in the research study. All subjects were normal controls. The reasons why they were referred to the center were mainly the presence of cognitive complaints, renewal of driver’s licenses, or as part of a general health check requested by their family doctor.

The instruments were administered through an individual structured interview lasting approximately 90 minutes. After data collection was completed, a written report was delivered to them. All subjects confirmed their willingness to participate in the study by signing informed consent.

### Data analysis

First, descriptive statistics for sociodemographic variables and cognitive performance were obtained. Then, Spearman rank correlation analysis was performed between occupational complexity, household tasks complexity, and cognitive measures. Finally, a path analysis was effectuated in order to analyze the incidence of occupational complexity and complexity of household tasks on measures of memory, executive functions, and language. In order to carry out the analysis of structural equations, the IBM Statistical Package for Social Sciences (SPSS) - Analysis of Moments Structure (AMOS) 22 program for Windows was used^31^.

## RESULTS

First, a sociodemographic description of the sample was made. The highest percentage was found in the female gender (68%). The mean age was 72.34 (standard deviation [SD]=5.07) and the educational level was 13.26 (SD=3.21). Regarding occupational complexity, the positions most occupied by participants before retirement were: administrative (32%), unskilled trades (21%) and merchants (21%), and senior executives (13%).

Table 1 shows the raw scores and their equivalent z-score values on neuropsychological tests. In their transformation to z-scores, it is observed that the average values obtained were higher than z=-1.5, which indicates an adequate performance for their age, sex, and education in the administered neuropsychological tests. When assessing the fit of variable distributions to a normal distribution, analyses conducted using the Shapiro-Wilk statistic indicated that all measures significantly deviated from the theoretical values of the normal distribution (p<0.001). For this reason, correlation analyses were conducted using the Spearman rank correlation coefficient.

**Table 1:** Cognitive performance in neuropsychological tests.

Test	Raw score		Z-score	
	M	SD	M	SD
Mini-state examination	28.36	1.70	0.33	0.78
Boston naming test	51.33	1.25	-0.47	1.19
Vocabulary WAIS III	45.22	1.34	0.37	0.72
Semantic fluency	18.32	4.87	-0.16	1.06
Phonological fluency	15.11	4.26	0.26	1.07
Delay recall logic memory	8.50	1.88	-0.46	1.52
Delay recall word list	8.22	2.41	-0.76	1.16
Cubes WAIS III	24.35	1.78	-0.11	0.75
Trail making test B	78.14	2.13	-0.68	1.08
Analogies WAIS III	16.36	1.78	-0.34	0.79
Digit backwards	5.46	1.23	0.42	1.20

Abbreviations: M, average; SD, standard deviation; WAIS III, Weschler Adult Intelligence Scale version III.

Table 2 reveals the Spearman rank correlation analysis performed between occupational complexity, household chores complexity, and cognitive measures obtained in memory, executive functions, and language.

**Table 2:** Correlations (rho) between occupational complexity and household tasks and cognitive measures.

	Occupational complexity	Complexity of household tasks
Memory		
Delay recall logic memory	0.177*	0.179*
Delay recall word list	0.136	0.024
Executive functions		
Digits backwards	0.320^†^	0.187*
Analogies WAIS III	0.410^†^	0.043
Cubes	0.324^†^	0.114
TMT-B	0.387^†^	0.145
Language		
Vocabulary WAIS III	0.456^†^	0.112
Boston Naming Test	0.048	0.094
Semantic fluency	0.171*	0.132
Phonological fluency	0.244^†^	0.126

Abbreviations: WAIS III, Wechsler Adult Intelligence Scale version III; TMT-B, Trail Making Test Part B.

Notes: Significance level

*p<0.050;

^†^p<0.010.

The results indicated a low-intensity correlation between household task complexity and delayed recall of logic memory (rho=0.17; p=0.031) and digits backwards (rho=0.18; p=0.027). On the other hand, occupational complexity showed significant associations of medium intensity with: the Digits Backwards Test (rho=0.32; p<0.000), Analogies WAIS III (rho=0.41; p<0.000), Cubes WAIS III (rho=0.32; p=0.000), TMT-B (rho=0.38; p=0.000), Vocabulary WAIS III (rho=0.45; p<0.000), and Phonological Fluency (rho=0.24; p=0.003). Likewise, it also showed low-intensity correlations with the measure of logic memory delayed recall (rho=0.17; p=0.033) and Semantic Fluency (rho=0.17; p=0.039).

In order to analyze the incidence of occupational complexity and the complexity of household chores on memory, executive functions, and language measures, a path analysis was executed. A model was tested in which occupational complexity and complexity of household chores (independent variables) directly influenced three latent factors: memory, executive functions, and language (dependent variables). The latent memory factor comprised delay recall logic memory and delay recall word list measures. The executive functions latent factor was composed of Digits Backwards, Analogies WAIS III, Cubes, and TMT-B measures. Meanwhile, the language latent factor was constructed from Vocabulary WAIS III, Boston Naming Test, Semantic Fluency, and Phonological Fluency measures. For this analysis, the maximum likelihood estimate was used as a matrix among the measures as input for the data analysis^31^, and the fit indices were used, following recommendations and conventions^32^. The fit indices chosen were GFI (Goodness of Fit Index), CFI (Comparative Fit Index), IFI (Incremental Fit Index), and RMSR (Root Mean Square Residual). The model proposed direct effects of the measures of occupational complexity and of complexity in household tasks on three latent factors. One of them is of memory (made up of measures of logic memory delayed recall and delayed recall of word list), another one of executive functions (made up of the measures of Analogies, Digits Backwards, TMT-B, and Cubes) and a third factor, language (made up of the measures of Vocabulary, Semantic Fluency, Phonological Fluency and the Boston Naming Test).

**Figure 1:**
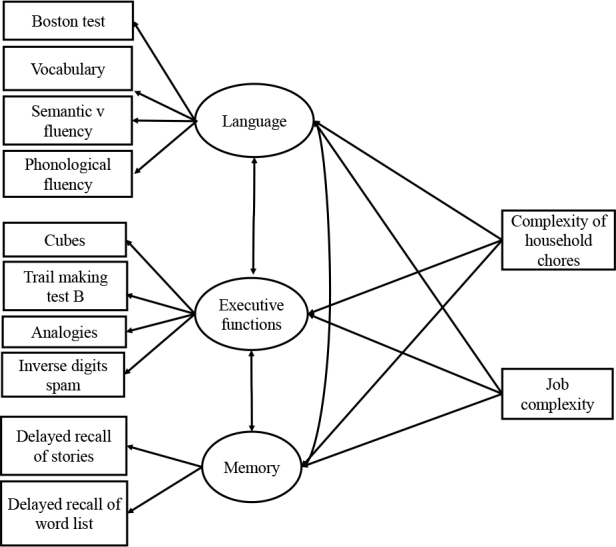
Model of the effect of occupational complexity and the complexity of household tasks on language, executive functions, and memory.

Figure 1 shows the proposed model of the effect of the cognitive reserve.

**Figure 2:**
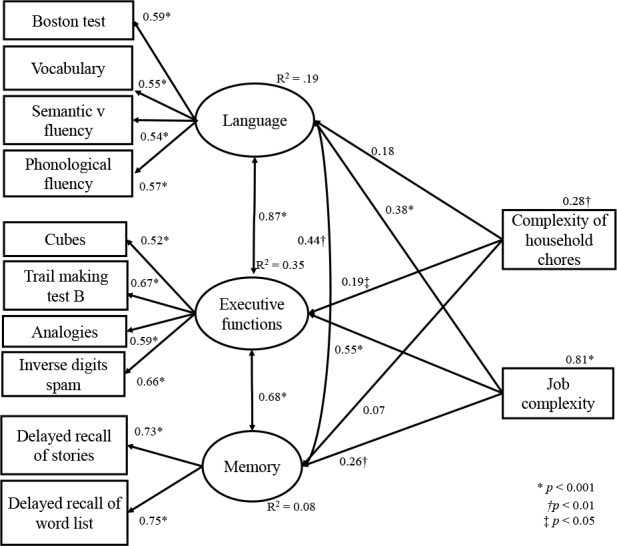
Factor loadings, correlations, regression weights, and the coefficient of determination of the model of the effect of occupational complexity and the complexity of household tasks on language, executive functions, and memory.

The analysis detected a good fit of the model to the data obtained (GFI=0.93; CFI=0.93; IFI=0.94; RMSR=0.05). When analyzing the regression weights, the model presented adequate and significant factor loadings, as well as significant correlations between memory, executive functions and language factors (Figure 2). Regarding the regression weights, it is observed that the complexity of household tasks showed significant effects on the executive functions factor (β=0.19; p=0.027), but not on the memory factors (β=0.07; p=0.435), or language (β=0.18; p=0.068). On the other hand, occupational complexity showed significant effects on the three factors: memory (β=0.26; p=0.008), language (β=0.38; p<0.001), and executive functions (β=0.55; p<0.001).

R2: coefficient of determination.

## DISCUSSION

The aim of the present study was to assess the impact of occupational complexity and household tasks on three cognitive domains (verbal episodic memory, language, and executive functions) in older adults residing within the community.

In this regard, it was observed that occupational complexity has a significantly greater impact on all the cognitive domains analyzed: verbal episodic memory, executive functions, and language. However, household activities only indicate low-intensity associations in some domains such as logical memory, working memory, and some aspects related to executive functions. Therefore, occupational complexity would be associated with better performance in various psychological functions. These results coincide with what was stated by Zhu et al.^33^ who indicate the risk to which workers who perform manual tasks are exposed compared to those who are inserted into more complex work environments and with interactions with different materials, especially data and people.

To the stated results, it is added that the conducted trail analysis detected a good fit of the model to the data obtained. When analyzing the regression weights, the model shows adequate and significant factor loadings, and important correlations between memory, executive functions, and language factors. Regarding the regression weights, it is observed that the complexity of household tasks shows considerable effects on the executive function factor, but not on memory or language factors. On the other hand, occupational complexity shows significant effects on the three factors. The results add evidence that occupational complexity would be a factor that promotes cognitive reserve, in contrast to household activities, which seem to have a moderate impact on cognition^34^. These results coincide with what is stated by Jiang & Xu^35^ who suggest that not only do the particularities of the complexity of the activity and its environment impact on the economic and social situation and possibilities, but it may also determine different courses of aging and cognitive risk. From this, it can be inferred that manual workers would be in a situation of greater vulnerability to develop pathologies that affect the cognitive sphere than those who have more complex and varied occupations considering the type of cognitive demand involved in the completion of the task.

Therefore, the results of this study provide elements of judgment of interest for professionals dedicated to the care of older adults and the planning of social policies, both public and private. These ones favor their social participation and general well-being. Within the framework of activity theory, paid work, among other factors, could exert a positive modulating effect on cognitive impairment. Regarding the results observed about the main occupations of the group of subjects evaluated, it is important to point out that most of them had jobs of intermediate complexity. The main occupations were administrative (32%), unskilled trades (21%) and merchants (21%), and senior executives (13%). Because complex jobs often require higher education, previous research^36,37^ suggests that higher education leads to greater occupational complexity, which in turn, protects cognitive functioning by cultivating cognitive reserve. Suemoto et al.^38^ emphasize the importance of education in the construction of cognitive reserve. Considering the sample interviewed and the values observed, it would be advisable for future studies to include larger and more heterogeneous samples in terms of work occupation, to examine the impact that the different jobs have on cognition.

Furthermore, it is suggested that future studies deepen the analysis of the impact that daily life activities have on the construction of cognitive reserve. This variable, as mentioned above, is often invisible in scientific studies that address this issue and is often not differentiated from leisure time activities. Johnson et al.^39^ in a previous study indicate that social participation in stimulating environments proves to be an environmental factor that can collaborate or act as a buffer against the appearance of diseases that affect cognition, such as Alzheimer’s disease and other dementias. Consequently, the application of intervention strategies in vulnerable populations of subjects who carry out mainly manual work would be of great value. It is in leisure time activities that these workers can have the opportunity to be exposed to more complex environments with different stimuli with which they interact in their paid jobs or household activities. Leisure time activities provide excellent opportunities for brain enrichment^40^. Therefore, a possible intervention strategy would be for government agencies or social or community organizations to offer alternative spaces where individuals can find activities rich in cognitive stimuli as an opportunity for mental training, recreation, and socialization. Currently, some activities like learning a second language or musical training are pointed out as promoting cognition in old age, and others, like attending art museums, can be framed in health practices and not only as leisure and recreation spaces. It is during leisure time activities that individuals can enrich their cognitive abilities, compensating for the stimulation not received due to the educational and job placement opportunities experienced^41^. Complementary, it would be interesting to have more studies with larger and more heterogeneous samples that deepen the analysis of the impact that home activities have on cognition and the formation of cognitive reserve. These activities also have a strength given that they are ecological and performed by most individuals. Knowing their impact on cognition can offer a powerful source of stimulation, especially for those who cannot access other types of activities apart from work.

However, the study on the subject should be continually deepened, especially to analyze the differences between men and women about the impact that domestic and work occupation have on cognition in individuals of different genders. It is suggested that future studies, with a larger number of subjects, can analyze the variables presented herein according to gender. An important limitation to mention is that it is a cross-sectional study that did not allow us to examine the influence of work occupation over time. It is suggested, for future studies, the application of the longitudinal data model that could provide new information on the impact of employment and housework on the process of decline in older adults with cognitive risk. Another limitation of the study is the lack of neuroimaging studies, genetic information, and other biomarkers, which could be useful in characterizing the modulating role of work occupation with respect to cognitive performance at this stage of life.

As a general conclusion, despite the limitations of the study, it can be stated that this first analysis provides, in part, answers to the main objective proposed. It provides valuable information for the local environment about the positive impact that paid work over housework has on older adults’ cognition, in addition to supplementary support for the idea that occupational complexity may be associated with better cognitive functioning in old age than housework. These results are consistent with clinical studies conducted in this regard for pathologies that affect cognition. Likewise, it constitutes empirical evidence for research in cognitive neurology and gerontology, which may be of interest to the clinical area. This permits to guide the design of interventions that could improve the cognitive performance of older adults and thus delay the appearance of symptoms of dementia, such as Alzheimer’s disease. Finally, when analyzing possible psychosocial factors that buffer cognitive performance in old age, elements of the judgment of interest would be applied to the area of old age psychology and neuropsychology. This would be accomplished through the incorporation of behaviors that promote healthy cognitive aging and the role of leisure time activities in a population vulnerable to developing cognitive risk, and the indication of disparities between those who perform paid work and manual or domestic work.
